# Combining antibody markers for serosurveillance of SARS-CoV-2 to estimate seroprevalence and time-since-infection – CORRIGENDUM

**DOI:** 10.1017/S0950268822001017

**Published:** 2021-11-25

**Authors:** Md S. Bhuiyan, Ben J. Brintz, Alana L. Whitcombe, Alena J. Markmann, Luther A. Bartelt, Nicole J. Moreland, Andrew S. Azman, Daniel T. Leung

**Affiliations:** 1 Division of Infectious Disease, University of Utah School of Medicine, Salt Lake City, UT, USA; 2 Division of Epidemiology, University of Utah School of Medicine, Salt Lake City, UT, USA; 3 Faculty of Medical and Health Sciences, University of Auckland, Auckland, New Zealand; 4 Maurice Wilkins Center, University of Auckland, Auckland, New Zealand; 5 Department of Medicine, Division of Infectious Diseases, University of North Carolina School of Medicine, Chapel Hill, NC 27599, USA; 6 Department of Epidemiology, Johns Hopkins Bloomberg School of Public Health, Baltimore, MD, USA; 7 Faculty of Medicine, Institute of Global Health, University of Geneva, Geneva, Switzerland; 8 Divsion of Microbiology & Immunology, University of Utah School of Medicine, Salt Lake City, UT, USA

The original article was published with an incorrect funding acknowledgement.

Currently, the funding statement reads,

This work was supported in part by the National Institutes of Health (R01 AI135115 to D. T. L.), with funding in part from the National Center for Research Resources and the National Center for Advancing Translational Sciences of the National Institutes of Health, through Grant UL1TR002538 (formerly 5UL1TR001067-05, 8UL1TR000105 and UL1RR025764), and from the SeroNet programme of the National Cancer Institute (1U01CA261277-01) and the NIH SeroNet Serocenter of Excellence Award (U54 CA260543).

It should read:

This work was supported in part by the National Institutes of Health (R01 AI135115 to D. T. L.), with funding in part from the National Center for Research Resources and the National Center for Advancing Translational Sciences of the National Institutes of Health, through Grant UL1TR002538 (formerly 5UL1TR001067-05, 8UL1TR000105 and UL1RR025764), and the NIH SeroNet Serocenter of Excellence Award (U54 CA260543).

This has now been updated in the original article.
